# Evaluation of Outpatients in the Post-COVID-19 Period in Terms of Autonomic Dysfunction and Silent Ischemia

**DOI:** 10.7759/cureus.40256

**Published:** 2023-06-11

**Authors:** Muammer Karakayalı, Inanc Artac, Dogan Ilis, Timor Omar, Ibrahim Rencuzogullari, Yavuz Karabag, Mehmet Altunova, Ayça Arslan, Ezgi Guzel

**Affiliations:** 1 Cardiology, Kafkas University School of Medicine, Kars, TUR; 2 Cardiology, Mehmet Akif Ersoy Thoracic and Cardiovascular Surgery Training Research Hospital, Kars, TUR

**Keywords:** post-acute covid-19 syndrome, coronary microvascular dysfunction, heart rate variability, ambulatory monitoring, covid-19

## Abstract

Introduction and objective: In this context, the objective of this study is to evaluate the 24-hour ambulatory electrocardiography (ECG) recordings, autonomous function with heart rate variability (HRV), and silent ischemia (SI) attacks with ST depression burden (SDB) and ST depression time (SDT) of post-COVID-19 patients.

Materials and methods: The 24-hour ambulatory ECG recordings obtained >12 weeks after the diagnosis of COVID-19 were compared between 55 consecutive asymptomatic and 73 symptomatic post-COVID-19 patients who applied to the cardiology outpatient clinic with complaints of palpitation and chest pain in comparison with asymptomatic post-COVID-19 patients in Kars Harakani state hospital. SDB, SDT, and HRV parameters were analyzed. Patients who had been on medication that might affect HRV, had comorbidities that might have caused coronary ischemia, and were hospitalized with severe COVID-19 were excluded from the study.

Results: There was no significant difference between symptomatic and asymptomatic post-COVID-19 patients in autonomic function. On the other hand, SDB and SDT parameters were significantly higher in symptomatic post-COVID-19 patients than in asymptomatic post-COVID-19 patients. Multivariate analysis indicated that creatine kinase-myoglobin binding (CK-MB) (OR:1.382, 95% CI:1.043-1.831; p=0.024) and HRV index (OR: 1.033, 95% CI:1.005-1.061; p=0.019) were found as independent predictors of palpitation and chest pain symptoms in post-COVID-19 patients.

Conclusion: The findings of this study revealed that parasympathetic overtone and increased HRV were significantly higher in symptomatic patients with a history of COVID-19 compared to asymptomatic patients with a history of COVID-19 in the post-COVID-19 period. Additionally, 24-hour ambulatory ECG recordings and ST depression analysis data indicated that patients who experienced chest pain in the post-COVID-19 period experienced silent ischemia (SI) attacks.

## Introduction

Many patients, including patients who do not have cardiovascular risk factors or a history of cardiac or arrhythmic diseases, present with complaints of chest pain and palpitations to the cardiology outpatient clinics during the period after coronavirus disease 2019 (COVID-19). Most patients who became infected with COVID-19 recover completely without sequela, while some continue to have diverse symptoms, including autonomic dysfunction, for longer than 12 weeks after recovery from COVID, a condition referred to as “post-COVID-19 syndrome” or “long-COVID-19 syndrome” unless there is an alternative diagnosis [1,2].

After having recovered from the acute phase of COVID-19, up to 91% of COVID-19 patients reportedly experience persistent fatigue, gastrointestinal symptoms, body aches, and brain fog, whereas up to 35% of the COVID-19 patients have persistent chest pain/burning or chest tightness even six months after having been diagnosed with COVID-19. These patients have been termed as “long haulers”. The cardiovascular diagnostic data of these long-haulers are usually normal and do not provide any information on the mechanism of the disease [3].

Heart rate variability (HRV) is a simple, non-invasive, objective, and validated parameter used in the assessment of autonomic nervous system function. The time-domain indices of HRV describe the amount of variation in time between consecutive heartbeats. Time domain indices include the standard deviation of normal to normal (NN) intervals (SDNN), root mean square of consecutive RR interval differences (rMSSD), and percentage of adjacent R-R intervals differing by more than 50 ms divided by the total number of R-R intervals (pNN50). The frequency domain indices of HRV include low frequency (LF), very low frequency (VLF), and high frequency (HF) bands in spectral analysis. Frequency domain indices of HRV include low-frequency (LF) and high-frequency (HF) bands in spectral analysis. The HF delineates the parasympathetic activity, whereas the LF delineates both sympathetic and parasympathetic activity, and SDNN, rMSSD, and pNN50 describe the parasympathetic activity. LF is the only indicator that assesses sympathetic activity. The LF/HF index is considered a measure of sympathetic and parasympathetic balance [4]. Impaired HRV has been associated with poor outcomes in various diseases [4]. The HRV triangular index, which is calculated by dividing the total number of NN intervals by the maximum density distribution, is the most frequently studied parameter. Lower values of the HRV triangular index were associated with increased mortality [5].

In the Poincaré plots, the SD1 width reflects the parasympathetic activity, and the SD2 length reflects the sympathetic modulation [6]. The shape of the Poincaré plot can be used to visually evaluate the sympathovagal activity. An elongated, torpedo-like shape with a decreased SD1/SD2 ratio is associated with an elevated sympathetic tone, and an oval, fan-shaped configuration resulting from an increased SD1/SD2 ratio is associated with reduced sympathetic tone [6].

It is known that COVID-19 may cause cardiac involvement at macrovascular and microvascular levels [7]. Acute cardiovascular manifestations of COVID-19 include myocarditis, ST-elevation myocardial infarction (STEMI), arrhythmias, vascular endothelial dysfunction, and coronary vasospasm [8]. Some patients report persistent symptoms after recovering from acute COVID-19. Chest pain is observed in 20% of patients with a history of COVID-19 [9], but the mechanisms for these symptoms have not been adequately elucidated. Mechanisms of microvascular disease observed in association with COVID-19 include endothelial injury with endothelial dysfunction and microvascular inflammation, and thrombosis [10].

Coronary microvascular angina (CMA) was reported in heterogeneous groups along with chest pain but without obstructive coronary artery disease (CAD), given that no objective measures of coronary flow reserve or endothelial function were obtained [11]. Prevalent ambulatory ischemia, predominantly silent ischemia (SI), which occurs at relatively low heart rates, was described in the literature on CMA. In a study that evaluated autonomic nervous system dysfunction by 24-hour electrocardiography (ECG) monitoring, one or more episodes of ST depression were observed in 14 (61%) of the 23 patients with CMA. Only one of these 14 patients had chest pain during the transient ST segment depression [12]. The presence of SI on ambulatory ECG monitoring has been associated with adverse cardiovascular outcomes, including death [13]. Anti-ischemic therapy directed at SI has reportedly improved outcomes in obstructive CAD patients [13].

In light of the foregoing, this study was carried out to evaluate the 24-hour ambulatory ECG recordings, autonomous function with HRV, and SI attacks with ST-depression burden (SDB) and ST depression time (SDT) of post-COVID-19 patients who applied to the cardiology outpatient clinic with complaints of palpitation and chest pain in comparison with asymptomatic post-COVID-19 patients.

## Materials and methods

Population and sample

The study population consisted of consecutive post-COVID patients evaluated in the outpatient clinic with 24-hour ambulatory ECG for indication of chest pain and palpitation during the post-COVID-19 period, i.e., within 12 to 26 weeks following the diagnosis of COVID-19. Patients with comorbidities that might have caused coronary ischemia, such as overt cardiovascular disease including CAD, arrhythmia, hypertension, left ventricular hypertrophy, moderate or severe valvular heart disease, renal and heart failure, morbid obesity, diabetes, positive exercise stress test, patients who have been on medication that might affect HRV, and patients who were hospitalized with severe COVID-19 were excluded from the study. In the end, 73 patients were included in the study sample. On the other hand, the control group consisted of 55 consecutive asymptomatic COVID-19 patients who had no palpitations, chest pain, cardiovascular disease or any related risk factors.

**Figure 1:**
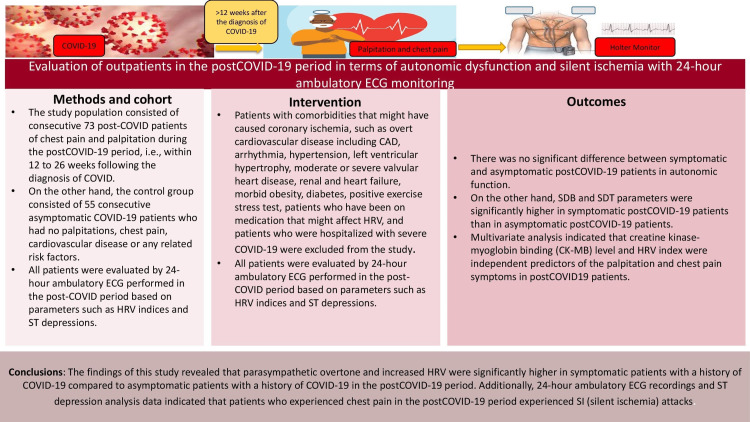
Graphical abstract.

All patients were evaluated by 24-hour ambulatory ECG performed in the post-COVID period based on parameters such as HRV indices and ST depressions. All cases included in the study had a history of COVID-19, confirmed by a real-time reverse transcriptase polymerase chain reaction (RT-PCR) test performed using nasopharyngeal swabs. None of the patients had any of the active COVID-19 signs and symptoms at the time of assessment and for a period of at least one month preceding the assessment. All patients’ medical comorbidities, physical examination findings, laboratory findings, and standard 12-lead ECGs obtained on the index day of outpatient visits were recorded. In addition, transthoracic echocardiography (ECHO) was performed for all patients using a Philips HD 11 XE ultrasound device (Andover, MA, USA). Patients with chest pain were evaluated with an exercise stress test according to risk analysis (see Appendix, Figure 1).

Ethics statement

The study protocol was approved by the local ethics committee (Ethics Committee of the Dean of the Faculty of Medicine of Kafkas University, 80576354-050-99/177 numbered ethics committee approval dated May 26, 2021).

Evaluation of heart rate variability and ST depression

Twenty-four-hour 12-lead ambulatory ECG monitoring was performed for all patients using a DMS300-4A Holter ECG recorder device (DM Software Inc., Beijing, China). ECG data were transferred from the recording unit (three-channel, double independent pacing channel BIOX Vasomedical CB-1303-C device, Vasomedical, Inc., Westbury, USA) to a computer using special software (CardioScan Premier 12, USA). Ambulatory ECG recordings were reviewed by two physicians masked to the subject's status for ST depression measured 80 ms away from the J point, with evidence of ≥ 1 minute horizontal or ≥ 1.0 mm down-sloping ST segment depression.

HRV was analyzed using 24-hour ambulatory ECG recordings through a multi-channel electronic data recording system that allows the transfer and analysis of ECG data. The recorded series of RR intervals was processed for frequency and time domain analysis over a 24-hour period. Additionally, frequency domain indices of HRV, including LF and HF, were calculated using spectral analysis over a 24-hour period. ECG-based evaluation of HRV may be performed by several methods, including time- and frequency-domain analyses as well as nonlinear techniques [14]. The analysis is usually based on long-term (at least 18 hours) Holter ECG recordings or short-term (usually five minutes) ECG recordings obtained under controlled standardized conditions to avoid any influence from external stimuli that may affect autonomic nervous tone.

Power of spectral components may be expressed in absolute (ms^2^) and normalized units (nu). Normalized units were obtained as follows: LF or HF norm (nu) = (LF or HF (ms^2^)) × 100/(total power (ms^2^) − VLF (ms^2^)). Five-minute recordings under controlled conditions are recommended for frequency domain analysis. It is assumed that at least one minute is needed to assess the HF component, while two minutes are required for LF analysis [15]. Spectral analysis may also be performed based on five-minute segments averaged over a 24-hour period yielding the averages of the modulations attributable to the LF and HF components. The Poincaré index (SD1, SD2, SD1/SD2 ratio) was computed.

ST depressions were evaluated in both symptomatic and asymptomatic groups. ST depressions between 08:00 AM and 12:00 AM were evaluated as daytime ST depressions, and ST depressions between 12:00 AM and 08:00 AM were evaluated as night-time ST depressions. Daytime and night-time ST depressions per hour were determined.

Statistical analysis

The normal distribution characteristics of the variables investigated within the scope of the study were evaluated using the Kolmogorov-Smirnov test. Continuous variables were expressed as either median and interquartile range or mean and standard deviation (SD) values depending on their normal distribution characteristics and compared using the t-test or the Mann-Whitney U test, as appropriate. Categorical variables were expressed as numbers (n) and percent (%) values and analyzed using Pearson’s chi-squared test or Fisher’s exact test. The results of the regression analysis were expressed as odds ratios (OR) within a 95% confidence interval (CI). Statistical Product and Service Solutions for Windows, Version 25.0 (SPSS 25.0, IBM Corp., Armonk, NY, U.S., 2017) software package was used to conduct the statistical analyses.

The resulting data were presented as n (%) or mean±SD. The probability (p) statistics of ≤ 0.05 were deemed to indicate statistical significance. Multicollinearity was assessed by eigenvalues and condition indices. Linearity was tested following the logarithmic transformation of each parameter.

The effect of all variables included in the major adverse cardiovascular and leg events (MACE) was determined by univariate analysis based on the odds ratios. The parameters found to be significant in univariate analysis, i.e., smoking status, pNN50, VLF, HRV index, glucose, creatine kinase-myoglobin binding (CK-MB), pro-B-type natriuretic peptide (proBNP), and total cholesterol levels were further analyzed using multivariate analysis. Backward conditional logistic regression analysis was used to determine the independent predictors of the symptoms.

## Results

A total of 128 patients with a history of COVID-19 were included in the study. Of these patients, 73 with complaints of chest pain and palpitation were included in the symptomatic group, and 55 were included in the asymptomatic group. The distribution of patients’ characteristics by groups is shown in Table 1. There was no significant difference between the groups in clinical characteristics, hematological and biochemical parameters, except for smoking status (3 (5.5%) vs. 3 (17.8%) active smokers, p=0.037) and pro-BNP level (37.52 (15.37-67.89) pg/ml vs. 18.32 (10.60-46) pg/ml, p=0.025).

**Table 1:** Distribution of demographic and laboratory characteristics by asymptomatic and symptomatic post-COVID-19 patient groups. COVID-19: coronavirus disease 2019, BMI: Body Mass Index, CAD: coronary artery disease, CRP: C-reactive protein, WBC: white blood cell, Htc: hematocrit, eGFR: estimated glomerular filtration rate, CK-MB: creatine kinase-myoglobin binding, pro-BNP: pro-B-type natriuretic peptide, LDL-C: low-density lipoprotein cholesterol, HDL-C: high-density lipoprotein cholesterol, TSH: thyroid-stimulating hormone, T3: triiodothyronine, T4: thyroxine, NS: not significant.

	Asymptomatic post-COVID-19 patient group (n:55)	Symptomatic post-COVID-19 patient group (n:73)	p-value
Age (years)	41±13	38±11	0.306
Gender, n (%) (female)	23(41.8)	41(56.2)	0.109
BMI (kg/m^2^)	26.16±3.66	25.57±4.76	0.138
Smoking status, n (%)	3(5.5)	13(17.8)	NS
Familial CAD history, n (%)	6(10.9)	7(9.6)	0.807
CRP (mg/L)	1.69(0.68–5.72)	1.82(0.80–3.50)	0.939
Albumin (mg/L)	43.52±6.44	43.73±4.53	0.261
Glucose (mg/dL)	104±31	94±21	0.055
Creatine (mg/dL)	0.74±0.24	0.74±0.18	0.686
Troponin (ng/mL)	6.40±3.08	5.99±2.92	0.260
Lymphocyte (10^3^/mL)	2.28(1.82–2.65)	2.29±0.79	0.878
WBC (10^3^/mL)	7.23±1.76	7.40±1.99	0.711
Hemoglobin (g/dL)	14.55±1.71	14.49±1.84	0.832
Htc (%)	43.45±4.81	43.73±4.85	0.743
Platelet (10^3^/mL)	269±74	280±61	0.337
Eosinophil (10^3^/mL)	0.11(0.06–0.16)	0.12(0.07–0.17)	0.336
eGFR (mL/min/1.73 m^2^)	105.09±17.42	107,88±17.40	0.227
CK-MB (U/L)	1.51(1.10–2.42)	2.27(1.25–3.64)	0.074
Pro-BNP (pg/mL)	37.52(15.37–67.89)	18.32(10.60–46)	0.025
Total cholesterol (mg/dL)	189±38	177±43	0.059
LDL-C (mg/dL)	111±37	101±38	0.113
HDL-C (mg/dL)	49±12	49±11	0.784
Triglyceride (mg/L)	143±64	147±72	0.952
TSH (mIU/L)	1.62(1.31–2.39)	1.55(2.35–2.27)	0.879
T3 (nmol/L)	3.38±0.59	3.11±0.61	0.893
T4 (nmol/L)	1.28±0.26	1.34±0.47	0.687

There was no significant difference between the groups in time domain indices of HRV analysis, i.e., SDNN, SDANN, rMMSD, and TP, whereas PNN50 was significantly higher in the symptomatic group than in the asymptomatic group (13.5 (7-23.2) vs. 18.9 (9-34), p=0.027). There was no significant difference between the groups in frequency domain indices of HRV, i.e., normalized LF power, LF, HF, and LF/HF values, whereas the VLF value was significantly higher in the symptomatic group than in the asymptomatic group (38052.87 (17395-105153.6) vs. 53969 (20367-178261.7), p=0.035). The HRV index was significantly higher in the symptomatic group than in the asymptomatic group (30±14 vs. 36±15, p=0.012) (Table 2). There was no significant difference between the two groups in terms of maximum heart rate (HR), minimum HR, average HR, maximum QT, maximum QTc, and >450 QTc (%) parameters (Table 2).

**Table 2:** Distribution of 24-hour ambulatory ECG rhythm monitoring findings by asymptomatic and symptomatic post-COVID-19 patient groups. HR: heart rate, QTc: corrected QT, SDNN: standard deviation of normal-to-normal R-R intervals 24-h, SDANN: standard deviation of the mean R-R intervals calculated over a five-minute period, rMSSD: square root of the mean squared difference of successive R-R intervals, pNN50: the percentage of the differences between adjacent normal R-R intervals exceeding 50 ms, TP: total power, VLF: very low frequency, LF: low frequency, HF: high frequency, HRV: heart rate variability, msn: milliseconds, bpm: beats per minute, min: minute, SD1: “width” of the Poincare plot, reflecting fast changes of the heart rate, SD2: “length” of the Poincare plot, reflecting slow changes of the heart rate, n: number.

	Asymptomatic post-COVID-19 patient group (n:55)	Symptomatic post-COVID-19 patient group (n:73)	p-value
Maximum HR, bpm	124±16	125±17	0.593
Minimum HR, bpm	47±6	48±6	0.294
Average HR, bpm	77±9	79±10	0.217
Max. QT, msn	437±33	436±42	0.391
Max. QTc, msn	478±31	479±42	0.204
>450 QTc, %	5.30(1.30–16.30)	5.08(1.57–21.01)	0.979
SDNN, ms	141.7±41	137.6±40.4	0.581
SDANN, ms	140.8±60.8	134.1±51.7	0.502
rMSSD, ms	60.7(34.9–90.1)	59.7(44.9–74.8)	0.686
PNN50, %	13.5(7–23.2)	18.9(9–34)	0.027
TP, ms^2^	28678.4(9372.8–65057.2)	29501.9(6048.2–65057.2)	0.900
VLF, ms^2^	38052.9(17395–105153.6)	53969(20367–178261.7)	0.035
LF, ms^2^	11153.7(5850.5–28344.9)	11363.7(8578.7–39295.1)	0.052
HF, ms^2^	4260.1(2017.8–9380.8)	4798.6(2661.4–12553.8)	0.195
LF/HF	2.87±0.92	3.02±1.50	0.881
HRV index	30±14	36±15	0.012
ST depression time (SDT), min	16.50(2.60–71.00)	54.10(4.30–143.00)	0.026
ST depression burden (SDB), mv	−1.70(−0.24 to −3.06)	−2.50(−1.10 to −6.19)	0.019
SDNN >60 ms, n (%)	19(34.5)	42(56.8)	0.013
RMSSD >40 ms, n (%)	17(30.9)	38(51.4)	0.021
Normalized LF	69.92(64.76–75.98)	71.60(−7.94 to 80.05)	0.591
Daytime ST depression (n per hour)	0.00(0.00–0.44)	0.25(0.00–0.75)	0.034
Night-time ST depression (n per hour)	0.13(0.00–1.00)	0.38(0.00–1.25)	0.149
SD1	14.02±7.21	19.83±4.94	<0.001
SD2	52.57±15.42	60.16±14.12	<0.001
SD1/SD2	0.28(0.15–0.39)	0.32(0.26–0.45)	0.012

To demonstrate the parasympathetic overtone, RMSSD >40 ms and SDNN >60 ms were compared between the groups. SDNN >60 ms (42 (56.8%) vs. 19 (34.5%), p=0.013) and RMSSD >40 ms (38 (51.4%) vs. 17 (30.9%), p=0.021) were more prevalent in the symptomatic group (Table 2). In line with these findings, the SD1 and SD1/SD2 ratios with increased parasympathetic activity were significantly higher in the symptomatic group than in the asymptomatic group. 

SDT (16.50 (2.60−71.00) vs. 54.10 (4.30−143.00), p=0.026) and SDB (−1.70 (−0.24 to −3.06)) vs. −2.50 ((−1.10 to −6.19), p=0.019) were significantly higher in the symptomatic group than in the asymptomatic group (Table 2). The number of ST depressions per hour in the daytime was significantly higher in the symptomatic group.

Variables found to be significant in the univariate analysis, i.e., pNN50, VLF, HRV index, glucose, CK-MB, pro-BNP, and total cholesterol levels, were further analyzed using the multivariate logistic regression analysis. Consequently, CK-MB (OR:1.382, 95% CI:1.043-1.831; p=0.024) and HRV index (OR: 1.033, 95% CI:1.005-1.061; p=0.019) were found as independent predictors of palpitation and chest pain symptoms in post-COVID-19 patients (Table 3).

**Table 3:** Results of the logistic regression models performed for symptomatic post-COVID-19 patients pNN50: the percentage of the differences between adjacent normal R-R intervals exceeding 50 ms, VLF: very low frequency, HRV: heart rate variability, CK-MB: creatine kinase-myoglobin binding fraction, pro-BNP: pro-B-type natriuretic peptide, OR: odds ratio, CI: confidence interval.

	Univariate analysis			Multivariate analysis		
	Univariate OR	95% CI	p	Multivariate OR	95% CI	p
pNN50	1.035	(1.005–1.067)	0.022			
VLF	1.000	(1.000–1.000)	0.049			
HRV index	1.033	(1.005–1.061)	0.019	1.045	(1.009–1.081)	0.013
Glucose	0.985	(0.970–1.000)	0.045			
CK-MB	1.382	(1.043–1.831)	0.024	1.933	(1.258–2.963)	0.003
Pro-BNP	0.993	(0.985–1.000)	0.049			
Total cholesterol	0.993	(0.984–1.000)	0.048			

## Discussion

This is the first study on the comparative analysis of symptomatic patients with chest pain and palpitation and asymptomatic patients by 24-hour ambulatory ECG during the post-COVID-19 period, that is, within 12 to 26 weeks following the patients were diagnosed with COVID-19, since a thorough literature review did not reveal any other study on the subject. The primary findings of this study are as follows: (i) HRV parameters such as PNN50, VLF, and HRV index were significantly higher in the symptomatic patients than in the asymptomatic patients, (ii) measures of parasympathetic overtone, i.e., SDNN >60 ms, RMSSD >40 ms, and SD1/SD2 ratio, were significantly higher in the symptomatic patients than in the asymptomatic patients, and (iii) parameters such as SDT and SDB were significantly higher in the symptomatic patients with chest pain than in the asymptomatic patients, and ST depression numbers were significantly higher during the daytime hours than in the night-time hours.

HRV is an autonomic nervous system parameter that can be measured in a simple, non-invasive way. The parasympathetic system increases HRV, while decreased vagal activity reduces HRV. HRV analysis is reportedly useful for the early detection of the acute inflammatory response and prognosis of COVID-19 in hospitalized patients [16].

Kaliyaperumal et al. found that acute COVID-19 infection was associated with parasympathetic dominancy [17]. Asarcikli et al. investigated autonomic functions in symptomatic patients with a history of COVID-19 during the post-COVID-19 period [16]. However, they included healthy individuals rather than asymptomatic post-COVID-19 patients with a history of COVID-19 in their control group, which they cited as a limitation of their study. In contrast, this study’s control group consisted of asymptomatic post-COVID-19 patients with a history of COVID-19, and HRV parameters such as PNN50, VLF, and HRV index were found to be significantly higher in the symptomatic group compared to the asymptomatic group. The total power of RR interval variability is the total variance and corresponds to the sum of the four spectral bands, LF, HF, VLF, and ultra-low frequency power (ULF). Spectral analysis obtained from short-term recordings is characterized by three major components, i.e., HF, LF, and VLF. While the vagal tone is considered to be a major contributor to the HF component, the exact pathogenesis of LF has not been fully elucidated. There are studies that propose that LF reflects the sympathetic system [18].

Coronary microvascular ischemia may be the cause of persistent chest pain in patients recovering from COVID-19. Once obstructive coronary disease has been ruled out, functional non-invasive testing is recommended to rule out myocardial ischemia. The pattern of recovery and the preferred management of post-COVID-19 microvascular dysfunction remain unresolved. A study reported that 27% of the patients evaluated in a COVID-19 unit had chest pain and that the chest pain in some of these patients were suggestive of angina. Although the pathological mechanism underlying this condition is still not fully clarified, microvascular dysfunction has been implicated in some studies [19]. 

A number of studies on obstructive CAD have demonstrated that ischemic attacks observed in outpatient monitoring independently predicted adverse outcomes [20]. In a two-year follow-up study conducted with 107 chronic stable angina patients, it was determined that 46 (43%) patients with one or more silent ischemia (SI) episodes had a three-fold increased risk of cardiac death compared to non-SI patients (24% vs. 8%, p=0.023) [21]. In comparison, in this study, evaluation of patients with chest pain in terms of obstructive CAD using the exercise stress test revealed significantly higher SDT and SDB in post-COVID-19 patients with chest pain than in post-COVID-19 patients without chest pain. Adverse outcomes resulting from SI in CMA patients have not been previously investigated with extensive controlled randomized trials (CRTs).

The relationship between COVID-19 and smoking and CAD has been well-established in the literature [22]. In line with the findings reported in the literature, the number of smoking patients was significantly higher in the symptomatic group than in the asymptomatic group in this study. Severe acute respiratory syndrome coronavirus 2 (SARS-CoV-2) enters epithelial cells through the angiotensin-converting enzyme-2 (ACE2) receptor [23]. Some evidence suggests that gene expression and subsequent receptor levels are elevated in the airway and oral epithelium of current smokers [22], thus putting smokers at a higher risk of contracting SARS-CoV-2 [24]. A number of studies have demonstrated that smoking impairs the autonomic nervous system [25], and causes chest pain and palpitations [25], due to cigarette components, which accelerate microvascular dysfunction [26]. Therefore, even though the results of the stress tests were normal in all patients included in this study, the effect of smoking on microvascular dysfunction cannot be ignored.

Circadian changes of ST depression have been demonstrated with continuous ECG monitoring in the literature [27]. However, none of these studies addressed post-COVID-19 patients. Hence, this is the first study to evaluate the circadian changes of ST depression in post-COVID-19 patients. The number of daytime ST depressions was significantly higher in symptomatic patients than in asymptomatic patients. Similarly, in another study, a significantly higher number of angina attacks were detected in patients during daylight hours, especially between 09:00 AM and 12:00 AM [27]. This can be attributed to the fact that morning hours during which individuals wake up and assume daily activities involve distinct physiological changes. Increases in blood pressure and heart rate during the morning lead to increased oxygen demand, whereas increased coronary vasotonus decreases the oxygen supply of the myocardium, resulting in a decreased ischemic threshold for angina pectoris [27].

Given that this is the first study to compare post-COVID-19 symptomatic and asymptomatic patients via HRV and ST depression analyses using 24-hour ambulatory ECG, its findings may provide guidance for further studies.

Limitations of the study

The single-center design of the study was its primary limitation. Secondly, the fact that only COVID-19 patients with mild to moderate disease severity were included in the study resulted in a relatively small sample size. Thirdly, given that coronary microvascular ischemia may be the cause of persistent chest pain in patients recovering from COVID-19, the lack of data on coronary microvascular dysfunction can be considered another limitation of this study.

## Conclusions

This study revealed that parasympathetic overtone and increased HRV were significantly higher in symptomatic patients with a history of COVID-19 than in asymptomatic patients in the post-COVID-19 period. Additionally, the results of the ST depression analysis conducted based on the 24-hour ambulatory ECG recordings demonstrated that patients with complaints of chest pain during the post-COVID-19 period experienced silent ischemia (SI) attacks. Future studies with longer outpatient follow-ups are needed to determine ST depression burden and ST depression time, elucidate the relationship between ST depression and adverse cardiovascular outcomes, and the mechanisms of coronary endothelial dysfunction and the related prognostic implications in COVID-19 patients.
